# Waterjet and laser etching: the nonlinear inverse problem

**DOI:** 10.1098/rsos.161031

**Published:** 2017-07-05

**Authors:** A. Bilbao-Guillerna, D. A. Axinte, J. Billingham, G. B. J. Cadot

**Affiliations:** 1 Machining and Condition Monitoring Group, Faculty of Engineering, University of Nottingham, University Park NG7 2RD, UK; 2 School of Mathematical Sciences, University of Nottingham, University Park NG7 2RD, UK

**Keywords:** waterjet milling, pulsed laser ablation, adjoint optimization, inverse problem

## Abstract

In waterjet and laser milling, material is removed from a solid surface in a succession of layers to create a new shape, in a depth-controlled manner. The inverse problem consists of defining the control parameters, in particular, the two-dimensional beam path, to arrive at a prescribed freeform surface. Waterjet milling (WJM) and pulsed laser ablation (PLA) are studied in this paper, since a generic nonlinear material removal model is appropriate for both of these processes. The inverse problem is usually solved for this kind of process by simply controlling dwell time in proportion to the required depth of milling at a sequence of pixels on the surface. However, this approach is only valid when shallow surfaces are etched, since it does not take into account either the footprint of the beam or its overlapping on successive passes. A discrete adjoint algorithm is proposed in this paper to improve the solution. Nonlinear effects and non-straight passes are included in the optimization, while the calculation of the Jacobian matrix does not require large computation times. Several tests are performed to validate the proposed method and the results show that tracking error is reduced typically by a factor of two in comparison to the pixel-by-pixel approach and the classical raster path strategy with straight passes. The tracking error can be as low as 2–5% and 1–2% for WJM and PLA, respectively, depending on the complexity of the target surface.

## 1. Introduction

Energy beam technologies such as waterjet and laser have emerged in research and industry as processes capable of controlled removal of mass from a wide variety of difficult-to-cut materials (ceramics, glasses, diamond-like composites, Ni/Ti alloys). These distinct machining techniques are usually studied and described separately, because they are based on very different material removal mechanisms. The size of the beam, which varies from macro- (waterjet milling, WJM) to micro- (pulsed laser ablation, PLA), defines the field of application [1,2]. However, there is one common aspect in all these processes, namely the effect of the dwell time of the beam against the target surface. For each technology, the mass removal rate depends mainly on the amount of time the beam stays at each position. This is an important difference to classical milling techniques, for which modelling of material removal is straightforward, and the main challenge consists of increasing productivity while maintaining stability. By contrast, modelling the removal rate in energy beam processes is more complex because the movement of the beam affects the shape of the etched surface.

Although they use different material removal mechanisms, WJM and PLA can both be described as time-dependent processes. WJM systems are usually employed for cutting or cleaning of a wide range of materials. In recent years, some studies have been presented that demonstrate their capability for milling. The process consists of a high velocity (up to twice the speed of sound), high-pressure jet of water which could contain abrasive particles (e.g. garnet, Al_2_O_3_) that is directed, by use of a focusing tube, at the target workpiece and removes material [3–5]; the smallest beam available has a diameter of around 0.2 mm. PLA systems can be used to remove much smaller amounts of material since beam diameters below 50 μm can easily be achieved. Moreover, PLA systems have higher precision and the dynamics of the machine have less influence than in WJM as the beam is usually directed by moving mirrors that have low inertial masses. However, the material can be damaged if the laser type and operating parameters are not properly selected [6,7].

WJM can be treated as a continuous process that depends on the instantaneous feed speed of the jet. By contrast, PLA processes are usually thought of as discrete systems and the total amount of material removed calculated by summing the effect of all the laser pulses. The overlapping of these pulses depends on the feed speed and the frequency between two consecutive shots. If the feed speed is sufficiently slow, consecutive shots are close enough that PLA can also be treated as a continuous process. This approach considerably reduces computation time and is more suitable for optimization purposes. Moreover, by treating PLA as a continuous process, a generic continuous time model can be used for both WJM and PLA, which leads to a common solution method for the inverse problem.

The direct problem is to determine the effect of the energy beam on the target workpiece surface for a given set of process parameters that describe the movement of the beam (the beam path). The inverse problem is to determine the control parameters, most importantly those which describe the beam path, that allow milling of a prescribed target profile (a freeform surface). The inverse problem is quite simple in conventional mechanical milling because the process is not time dependent and the geometry is well defined by the solid cutting tools. By contrast, the energy beam processes are time dependent and the trajectory of the beam has a large influence on the final etched surface.

Two main approaches have been used to solve the direct problem for WJM processes. Many studies use an analytical/geometrical approach to describe the behaviour of these systems [8–11]. Other approaches use numerical methods [12–15]. The direct problem for PLA processes can also be solved by using analytical/geometrical approaches [16,17] or by modelling the physics of material removal [18–21]. Analytical approaches are of more practical use for the inverse problem as they require less computation time and are easier to implement in an optimization algorithm.

Although the inverse problem in a time-dependent process has been studied before [22], the strategy that was developed is simply to vary the dwell time of the beam at a sequence of pixels on the required surface. This is simply the leading-order approximation to the necessary strategy when the radius of the beam is small compared with the size of the feature that is being etched. There have been some studies of the inverse problem for other time-dependent processes: electrochemical machining [23], where the tool/electrode works in tangential mode to envelop the required surface and is more similar to the movement of a cam than to the inverse problem of a time-dependent process; electro-discharge machining [24] where the electrode, with wear pattern measured at regular time intervals, copies the geometry of the final surface, so a mathematical solution of the inverse problem is not required. A solution of a reduced version of the inverse problem for WJM was presented in [25] using a frequency domain approach. However, the solution was only valid when single and multiple trenches with small values of the overlapping were generated. A linear model is only accurate enough to describe the behaviour of the process when the angle of incidence is approximately constant during the etching process. Moreover, the solution was restricted to one spatial dimension; most waterjet milling is performed outside this restrictive domain.

The objective of this paper is to present a practical method of solving the inverse problem for nonlinear machining (overlapping trenches) valid for both WJM and PLA in two spatial dimensions, thus making possible the generation of high-precision full freeform surfaces. Frequency-domain analysis [25] is no longer a helpful tool since this approach cannot be used with a nonlinear model. Instead, we numerically solve a partial differential equation model of the process and seek to determine the parameters that control the path of the beam by minimizing a cost function based on the desired freeform surface. We use a discrete adjoint algorithm to cheaply determine the Jacobian, along with a simple gradient-based method to find a local minimum, starting from an initial guess based on overlapping straight passes with dwell times determined pixel by pixel (the standard approach). Adjoint optimization is often used in parameter identification problems [26,27]. The main advantage of the adjoint algorithm is that it requires significantly less computation time than finite difference approaches to calculating the Jacobian. This is very useful when a large number of parameters need to be identified, as is the case for the inverse problem, where the feed speed of the beam has to be calculated at a large number of points for each pass in order to obtain a good solution. Note that we do not claim to have identified a globally optimal solution of the inverse problem; this is probably NP-hard given the space of possible beam paths that are available and techniques for generating solutions that use more complex paths ( for example, Peano curves, travelling salesman paths or spirals) are the subject of current work. The technique that we propose here is tested for the two processes (WJM, PLA) studied in this paper, but it could be applied to any time-dependent material removal process defined by the generic nonlinear model. The theoretical and experimental results show that the tracking error of the target freeform surface can be reduced by around a factor of two compared to the standard pixel-by-pixel technique.

Finally, note that the dynamics of the machine often need to be considered in the calculation of the trajectory of the beam. In some machining devices, the user can design the parameters of the controller and be able to (partially) compensate for the machine dynamics. However, sometimes these parameters are not known or cannot be changed. Then, a simple solution can be applied to compensate this effect on the selection of the slope of a step profile [28]. When the dynamics of the machine cannot be identified and compensated, the approach presented here can still be used if the target surface is selected in such a way that high-frequency components are avoided [25].

## 2. Generic model of an energy beam process

The generic, partial differential equation model used in this paper is

2.1 $$\frac{\partial Z}{\partial t}=-E(r;\text{a})\;f(\nabla Z,|\text{V}|,t;\text{u},\text{a}).$$

In this model, *E* is the etching rate function or beam footprint, *t* is time with 0≤*t*≤*T*, *z*=*Z*(**x**,*t*) is the evolving position of the surface in a Cartesian coordinate system (*x*,*y*,*z*), where **x**=(*x*,*y*) is spatial position in the *xy*-plane, ∇*Z*(**x**,*t*) is the orientation of the surface, **a** is a vector of constant parameters that characterize the model for each process ( for example, beam diameter, pump water pressure in WJM, pulse duration in PLA) and *r*=|**x**−**X**(*t*;**u**)| is the distance to the centre of the beam projected onto *z*=0. The centre of the beam is at **x**=**X**(*t*;**u**)=(*X*(*t*;**u**),*Y* (*t*;**u**)), **V**(*t*;**u**)=∂**X**(*t*;**u**)/∂*t* is the velocity of the beam, |**V**| is the feed speed and **u** is a vector containing the path control parameters. The appearance of ∇*Z* in *f* indicates that, in general, the slope of the evolving surface affects the rate at which material is removed. The possible nonlinear influence of the movement of the beam is also included in the model since *f* depends on |**V**|. The intervals of the operating parameters in **a** and the range of values of the feed speed for each process were chosen based on previous investigations, in such a way that workpiece surface damage/defects are avoided. For this reason, low values of the feed speed are not used.

In the forward problem, the final surface is determined by the choice of **u**, which specifies the path of the beam. In the inverse problem, the objective is to find **u** given a specified final surface, *Z*^target^(**x**). Note that the etching rate function only depends on the value of *r* for a given vector **a**, while the rest of the variables are included in a nonlinear function *f*. If all these variables have no influence on the behaviour of the beam, then *f*=1 and (2.1) becomes a linear model [25].

### 2.1. Model of waterjet milling

It is well known that the etched depth in WJM depends on the angle between the jet and the surface [10]. When shallow single trenches are etched, this angle is approximately constant during the process and a linear model can be used [25]. However, when deeper surfaces are etched, the angle of impact varies during the process (including across the beam) and its influence has to be taken into account. A nonlinear model of WJM is

**Figure 1.**
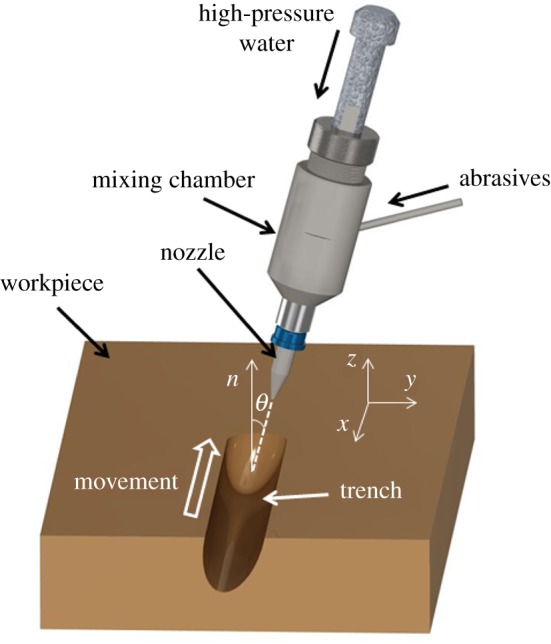
Continuous generation of a single trench using a WJM system.

2.2 $$\frac{\partial Z}{\partial t}=-E(r)f(\theta ),$$

where *θ* is the angle between the normal to the surface and the orientation of the beam defined by the unit vector **b** (figure 1). An outward normal to the surface is

2.3 $$\text{n}=\left(-\frac{\partial Z}{\partial x},-\frac{\partial Z}{\partial y},1\right),$$

and the angle between the jet and the normal can be obtained from

2.4 $$\cos\theta =\frac{\text{b}\cdot \text{n}}{\sqrt{1+{\left(\partial Z/\partial x\right)}^{2}+{\left(\partial Z/\partial y\right)}^{2}}}.$$

### 2.2. Model for pulsed laser ablation

**Figure 2.**
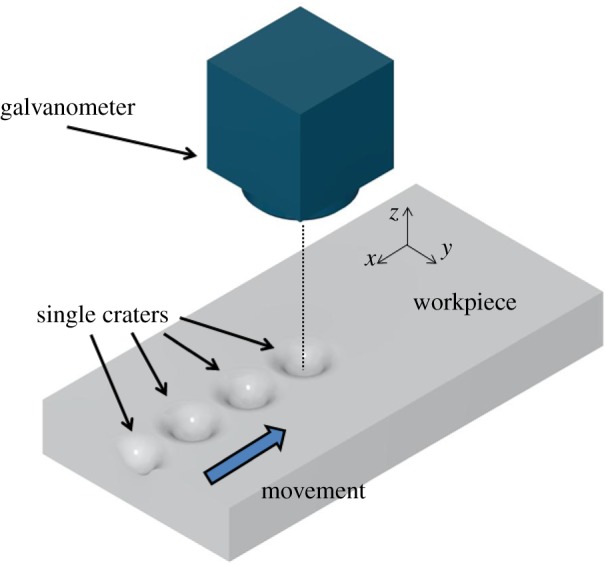
Discrete generation of the etched surface using a PLA system.

Although PLA processes are usually described using discrete pulse models, and the etched surface calculated by adding the effect of all the pulses shot during the process (figure 2), when the feed speed of the beam is sufficiently slow that consecutive shots overlap, the effect on the surface can be approximated as a continuous process [17]. Under this condition, a similar nonlinear model of PLA is

2.5 $$\frac{\partial Z}{\partial t}=-E(r)\;f(|\text{V}(t)|).$$

This continuous-time model can be solved significantly more efficiently than classical discrete models, where the effect of all the shots on the surface has to be calculated; this makes a continuous-time model more suitable for optimization purposes. The main limitation of the model is that it is only valid when consecutive shots overlap and cannot be used to predict single craters. However, freeform surfaces cannot be generated efficiently from single, non-overlapping craters, so we are not concerned with this restriction here.

## 3. Calibration and validation of the nonlinear model

### 3.1. Calibration of the waterjet milling model

The etching rate function, *E*(*r*), is calibrated experimentally using the technique described in [10], and we briefly describe the procedure here. A single trench needs to be generated with the jet perpendicular to the surface and feed speed high enough that the slope of the surface is small (a shallow trench). The etching rate function, *E*(*r*), can then be calculated using

3.1 $$E(r)=\frac{1}{\pi }\left[\int_{r}^{1}r\frac{Z_{f}(s)-Z_{f}(r)}{{\left(s^{2}-r^{2}\right)}^{3/2}}\;ds-\frac{Z_{f}(r)}{\sqrt{1-r^{2}}}\right],$$

where *Z*_*f*_ is the measured profile across the trench (averaged along the trench to minimize the effect of process noise) and the distance is measured in units of beam radius. In order to calibrate *f*(*θ*), which has been found to be well described by

3.2 $$f(\theta )=1+a_{1}\theta +a_{2}\theta^{2},$$

a second single trench must be generated, with the feed speed changing linearly between two values. Note that for shallow surfaces *θ* is small and *f* is close to 1. This leads to the linear model where only the etching rate function is important [25].

#### 3.1.1. Experimental set-up and calibration

**Figure 3.**
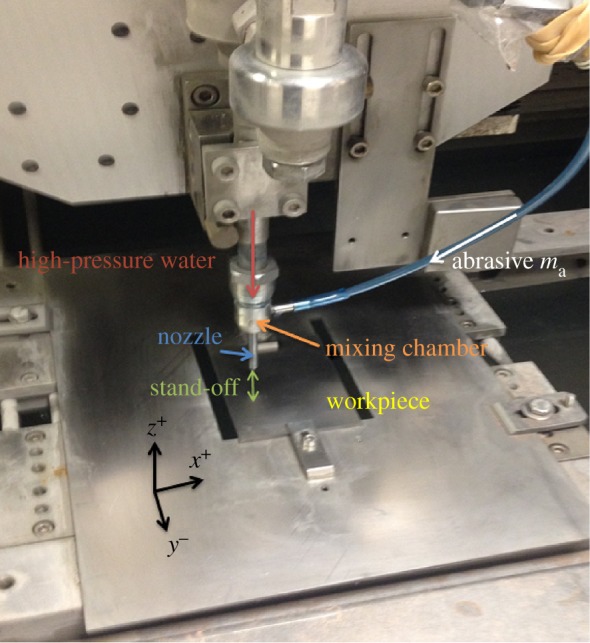
Experimental set-up for WJM.

The experimental data for model validation was generated with a Microwaterjet 3-axis F4 type (Waterjet AG, www.waterjet-group.com; figure 3) where various diameters of orifices (0.08–0.24 mm) and of focusing tube (0.2–0.8 mm) can be employed. The machine is empowered by a KMT streamline SL-V100D ultra-high pressure pump, capable of delivering pressures from 700 to 4000 bar. The apparatus was designed for high accuracies in jet positioning (less than 0.003 mm) and cutting (less than 0.01 mm) at maximum traverse speeds of 65 mm s^−1^. The abrasive particles used for this study were BARTON HPX 220, with pump pressure 138 MPa, grit mass flow rate $0.03\;\mathrm{kg}\;\min^{-1}$, and nozzle stand-off distance 3 mm for a combination of 0.18 mm and 0.5 mm diameters of orifice and focusing tube, respectively. The workpiece material was Ti6Al4V alloy, which is extensively used for the manufacture of aerospace and medical components. Values of the feed speed lower than 5 mm s^−1^ were avoided, so that no defects were generated. The resulting surfaces were measured using a white light interferometer (Bruker GT-i). The lateral resolution is 0.9 μm with the highest magnification (10×) and the vertical resolution is 0.1 nm. The maximum measurable slope is 70^°^.

In order to minimize the effect of pump and process noise in the calibration process, several trenches were generated using the same conditions and the mean profile measured. The first, shallow trench was generated with a jet feed speed of 60 mm s^−1^. The second, deeper trench was generated by varying the feed speed linearly from 8 to 65 mm s^−1^, which gave *a*_1_=0.5540 rad^−1^ and *a*_2_=0.1363 rad^−2^ in (3.2).

### 3.2. Calibration of the pulsed laser ablation model

A similar calibration for the etching rate function, *E*(*r*), is used for PLA, but several shallow trenches need to be generated, the average profile across each trench measured and *E*(*r*) for each obtained from (3.1). In this case, the function *f* is found to be dependent on the feed speed through

3.3 f=f(V(t))=α+β|V(t)|.

The parameters *α* and *β* can be determined using the data from these trenches, a process explained in detail in [17].

#### 3.2.1. Experimental set-up and calibration

**Figure 4.**
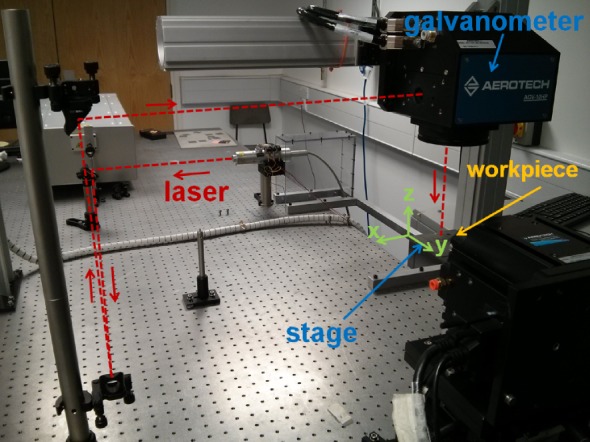
Experimental set-up for the PLA system.

The experimental tests were conducted with a SPI-G3 HM fibre laser with a constant pulse repetition rate that can be chosen between 1 and 35 kHz. The beam is fed directly into the galvanometer head, and an *f*-*θ* lens, of 100 mm focal length, is used to focus the beam onto the workpiece placed on the stage (figure 4). The resulting spatial profile has a beam width of around 45 μm with an ellipticity of 0.956 at the focal plane, where the maximum measured power is 18.8 W. For all experimental trials, the sample was positioned using a custom Aerotech system with a two-dimensional galvanometer head and a four-axis stage. This set-up is designed for high precision micro-machining, as the galvanometer offers an accuracy of 1 μm over the whole field of view. The sample positioning stage also offers 1 μm accuracy in the focal plane. To avoid distortion of the laser beam spot due to the angle introduced by the *f*-*θ* lens, the tests were carried out in the 1 mm^2^ at the centre of the field of view. The workpiece material used for the experiments was graphite. The machined surfaces were measured using the same white light interferometer as described in the previous section for WJM.

The first step is to find the range of values of feed speed, |**V**|, for which the continuous time model is valid: |**V**|>800 mm s^−1^ leads to insufficient overlapping of consecutive pulses, while |**V**|<200 mm s^−1^ produces deep trenches that are difficult to measure and damage the material. Several values of the frequency between consecutive pulses and the power were used in the study. In §5, the results obtained with a frequency of 35 kHz and the power 80% of the maximum are presented. The calibration proceeds as described above, and in more detail in [17].

## 4. Solution of the inverse problem

Now that the forward problem (i.e. for a given beam path, we can accurately predict the resulting processed surface) has been presented, in this section the solution of the inverse problem is presented (i.e. given a required final freeform surface, determine how to move the beam to obtain it).

### 4.1. Choice of beam path

**Figure 5.**
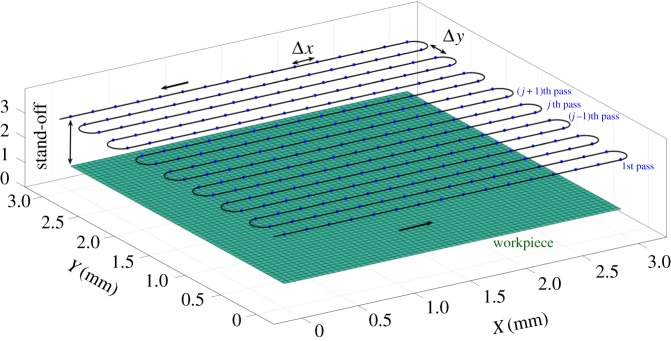
Example of parallel straight line paths (i).

**Figure 6.**
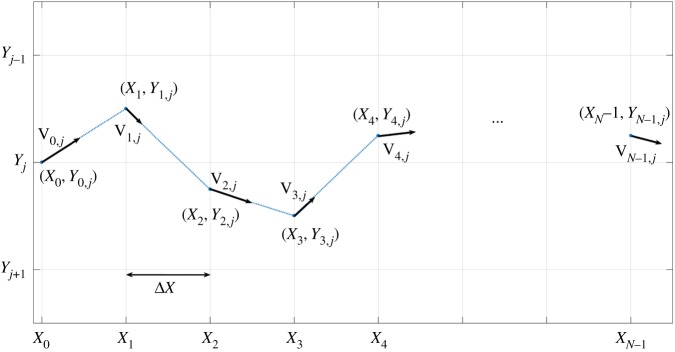
Path generation for each *y*-axis perturbed pass (ii).

Although it is reasonable to suppose that the optimal beam path (position of the centre of the beam) for etching a complex, freeform surface is itself complex, the automatic generation of such a path is a hard optimization problem. This process can be simplified if some constraints are introduced in the choice of the beam path. In this paper, two similar variations of the classical raster paths technique are chosen to define the beam path: (i) parallel straight line paths in the *x*-direction with constant overlap, Δ*Y*_jet_, in the *y*-direction (raster paths), parametrized by fixed points with equally spaced *x*-coordinates (figure 5), (ii) perturbed, parallel straight line paths, parametrized by points with equally spaced *x*-coordinates and perturbed *y*-coordinates (see figure 6, and figure 22 for a real example). In the former case, just the dwell time at each defining point is calculated, while in the latter, the free *y*-coordinate is also calculated, thereby doubling the size of the optimization problem. These extra degrees of freedom allow the beam path to match the shape of the prescribed freeform surface more closely.

For the second case, the control vector **u** is

4.1 $$\text{u}=[Y_{0,0},D_{0,0},Y_{1,0},D_{1,0},Y_{2,0},D_{2,0},\ldots ,Y_{N_{u}-1,N_{p}-1},D_{N_{u}-1,N_{p}-1}],$$

where *N*_*p*_ is the number of passes, *N*_*u*_ the number of points in each pass, *Y*_*i*,*j*_ the position of the centre of the jet in the *y*-direction and *D*_*i*,*j*_=|*d***X**_*i*,*j*_/d*t*|^−1^=|**V**_*i*,*j*_|^−1^ is the dwell time as a function of position along the path. The first subscript 0≤*i*≤*N*_*u*_−1 denotes the (*i*+1)th pixel for each pass, while the second subscript 0≤*j*≤*N*_*p*_−1 indicates the (*j*+1)th pass. The distance between consecutive points is chosen to be constant in the *x*-direction, so that

4.2 $$X_{i}=X_{i,j}=X_{i-1,j}+\Delta X\;\text{if }1<i\leq N_{u}-1,\;X_{0,j}=X_{0}\;\forall j.$$

Linear interpolation of dwell time between the pixels is used to determine **X**(*t*).

### 4.2. Discrete adjoint optimization algorithm

The trajectory of the beam only depends on the control parameters defined in (4.1), which parametrize **X**=**X**(*t*;*u*), so that

4.3 $$\frac{\partial Z}{\partial t}=-f(\nabla Z,D)E(|\text{x}-\text{X}(t;\text{u})|)=\frac{\partial Z}{\partial s}\frac{\partial s}{\partial t}=\frac{\partial Z}{\partial s}\frac{1}{D(\text{u})},$$

and hence,

4.4 $$\frac{\partial Z}{\partial s}=-D(s,\text{u})\;f(\nabla Z,D)E(|\text{x}-\text{X}(t;\text{u})|),$$

where s is arc length through the trajectory of the beam. It is more convenient to work with arc length and treat the beam position as a function of s, so we write **X**≡**X**(s;**u**). We will use a simple finite difference method to solve (4.4) (and its adjoint), with

4.5 $$\frac{Z_{n,m}^{k+1}-Z_{n,m}^{k}}{\Delta s}=-D_{k}f_{n,m}^{k}E_{n,m}^{k}$$

for 0≤*n*≤*N*−1, 0≤m≤*M*−1 and 1≤*k*≤*K*, where

4.6 $$\begin{array}{rl} E_{n,m}^{k} & =E(r_{n,m}^{k})=E(|{\text{x}}_{n,m}-\text{X}(s_{k},\text{u})|), \end{array}$$

4.7 $$\begin{array}{rl} {\text{x}}_{n,m} & =(x_{n},y_{m})=(n\Delta x,m\Delta y), \end{array}$$

4.8 $$\begin{array}{rl} Z_{n,m}^{k} & =Z({\text{x}}_{n,m},s_{k}), \end{array}$$

4.9 $$\begin{array}{rl} f_{n,m}^{k} & =f(\nabla Z_{n,m}^{k},D_{k}) \end{array}$$

4.10 $$\begin{array}{rl} \;\text{and}\;D_{k} & =D(s_{k},\text{u}). \end{array}$$

**Figure 7.**
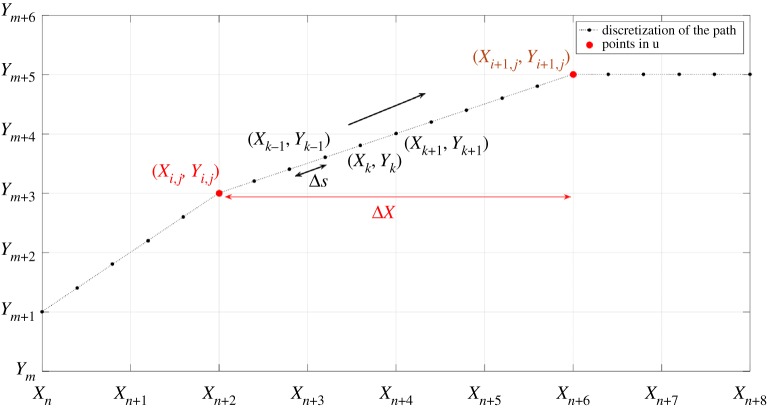
Discretization of the trajectory of the centre of the beam.

The subscripts *n* and m index the grid points in the *x*- and *y*-directions, with fixed grid spacings, Δ*x* and Δ*y*. The index *k* indicates the discretization of the position of the centre of the beam through the trajectory defined by **u**, Δ*s* is the distance between two consecutive points in the discretization of the trajectory through the same pass of the jet, so that s≡*kΔs*, $Z_{n,m}^{k}$ is the depth of the surface at **x**_*n*,m_ when the beam is at **x**=**X**(s_*k*_,**u**), and similarly for $f_{n,m}^{k}$ and $E_{n,m}^{k}$. The grid size is chosen to be smaller than the diameter of the beam in order to resolve its structure, and Δ*s* is chosen to be sufficiently small to resolve the highest frequency components of the trajectory of the beam. In practice, the available memory in the computer leads to a lower limit on the grid size. In this paper, this value was chosen to be between 10 and 20 times smaller than the diameter of the beam (figure 7). The discrete cost function is

4.11 $$J(\text{u})=\sum_{n=n_{1}}^{n_{2}}\sum_{m=m_{1}}^{m_{2}}{\left(Z_{n,m}^{K}-Z_{n,m}^{\mathrm{target}}\right)}^{2},$$

where *Z*^target^ is the target surface. The indices 0≤*n*_1_≤*n*_2_≤*N*−1 and 0≤m_1_≤m_2_≤*M*−1 denote the size of the target surface in the *x*–*y* plane. Note that the target surface is defined in a region slightly smaller than the full domain of solution, as this avoids using the beginning and end of each pass on the target surface, where the machine needs to start or stop its movement and the influence of the dynamics is more important. The optimization problem consists of finding u* that minimizes *J*, so that

4.12 $$J({\text{u}}^{*})\leq J(\text{u})\;\forall \text{u}\in S_{u},$$

where s_*u*_ is the set of possible control parameters.

The corresponding discrete Lagrangian is

4.13 $$L=J-\sum_{n=n_{1}}^{n_{2}}\sum_{m=m_{1}}^{m_{2}}\sum_{k=1}^{K}\left[H_{n,m}^{k}\left(\frac{Z_{n,m}^{k+1}-Z_{n,m}^{k}}{\Delta s}+D_{k}E_{n,m}^{k}f_{n,m}^{k}\right)\right]\Delta s,$$

where *H* is the Lagrange multiplier. The only coefficient of $Z_{n,m}^{K+1}$ in (4.13) is $H_{n,m}^{K}$, so $\partial J/\partial Z_{n,m}^{K+1}=0$, and hence

4.14 $$H_{n,m}^{K}=0\;\forall n,m.$$

Also, note that,

4.15 $$\frac{\partial J}{\partial Z_{n,m}^{k}}=\left\{ \begin{array}{ll} Z_{n,m}^{K}-Z_{n,m}^{\mathrm{target}} & \text{if}\;m=M-1,\\ 0 & \text{if}\;m<M-1. \end{array}\right.$$

For *n*_1_≤*n*≤*n*_2_ and m_1_≤m≤m_2_,

4.16 $$\begin{array}{rl} \frac{\partial L}{\partial Z_{n,m}^{k}} & =\frac{\partial J}{\partial Z_{n,m}^{k}}-[\frac{H_{n,m}^{k-1}-H_{n,m}^{k}}{\Delta s}+D_{k}\{\frac{E_{n-1,m}^{k}H_{n-1,m}^{k}{\left(\;f_{c}\right)}_{n-1,m}^{k}\nabla_{x}Z_{n-1,m}^{k}}{\Delta s}\\ & \;-\frac{E_{n+1,m}^{k}H_{n+1,m}^{k}{\left(\;f_{c}\right)}_{n+1,m}^{k}\nabla_{x}Z_{n+1,m}^{k}}{\Delta s}+\frac{E_{n,m-1}^{k}H_{n,m-1}^{k}{\left(\;f_{c}\right)}_{n,m-1}^{k}\nabla_{x}Z_{n,m-1}^{k}}{\Delta s}\\ & \;-\frac{E_{n,m+1}^{k}H_{n,m+1}^{k}{\left(\;f_{c}\right)}_{n,m+1}^{k}\nabla_{x}Z_{n,m+1}^{k}}{\Delta s}\}]\Delta s=0, \end{array}$$

where

4.17 $$\begin{array}{rl} c_{n,m}^{k} & =|\nabla Z_{n,m}^{k}{|}^{2}={\left(\nabla_{x}Z_{n,m}^{k}\right)}^{2}+{\left(\nabla_{y}Z_{n,m}^{k}\right)}^{2}, \end{array}$$

4.18 $$\begin{array}{rl} \nabla_{x}Z_{n,m}^{k} & =\frac{Z_{n+1,m}^{k}-Z_{n-1,m}^{k}}{2\Delta s}, \end{array}$$

4.19 $$\begin{array}{rl} \nabla_{y}Z_{n,m}^{k} & =\frac{Z_{n,m+1}^{k}-Z_{n,m-1}^{k}}{2\Delta s} \end{array}$$

4.20 $$\begin{array}{rl} \;\text{and}\;{\left(\;f_{c}\right)}_{n,m}^{k} & =\frac{\partial f}{\partial c_{n,m}^{k}}. \end{array}$$

Using (4.14) and (4.15) in (4.16) gives

4.21 $$H_{n,m}^{K-1}=\left\{ \begin{array}{ll} Z_{n,m}^{K}-Z_{n,m}^{\mathrm{target}} & \text{if }n_{1}\leq n\leq n_{2}\text{ and }m_{1}\leq m\leq m_{2},\\ 0 & \text{if }1\leq n<n_{1},\;n_{2}<n\leq N,\;1\leq m<m_{1}\text{ or }m<m\leq M. \end{array}\right.$$

Note that $H_{n,m}^{k}=0$ ∀*n*,m,*k* if the solution is a perfect fit.

For *k*<*K*, *n*_1_≤*n*≤*n*_2_ and m_1_≤m≤m_2_,

4.22 $$\begin{array}{rl} \frac{H_{n,m}^{k-1}-H_{n,m}^{k}}{\Delta s} & =D_{k}(\frac{E_{n-1,m}^{k}H_{n-1,m}^{k}{\left(\;f_{c}\right)}_{n-1,m}^{k}\nabla_{x}Z_{n-1,m}^{k}}{\Delta s}-\frac{E_{n+1,m}^{k}H_{n+1,m}^{k}{\left(\;f_{c}\right)}_{n+1,m}^{k}\nabla_{x}Z_{n+1,m}^{k}}{\Delta s}\\ & \;+\;\frac{E_{n,m-1}^{k}H_{n,m-1}^{k}{\left(\;f_{c}\right)}_{n,m-1}^{k}\nabla_{x}Z_{n,m-1}^{k}}{\Delta s}-\frac{E_{n,m+1}^{k}H_{n,m+1}^{k}{\left(\;f_{c}\right)}_{n,m+1}^{k}\nabla_{x}Z_{n,m+1}^{k}}{\Delta s}). \end{array}$$

For 1≤*n*<*n*_1_ , *n*_2_<*n*≤*N* and 1≤m<m_1_, m_2_<m≤*M*,

4.23 $$H_{n,m}^{k}=0\;\forall k.$$

Finally, ∂*L*/∂*u*_*i*_ leads to

4.24 $$\frac{\partial J}{\partial u_{i}}=\sum_{n=n_{1}}^{n_{2}}\sum_{m=m_{1}}^{m_{2}}\sum_{k=1}^{K-1}H_{n,m}^{k}\;f_{n,m}^{k}D_{k}\frac{\partial E_{n,m}^{k}}{\partial u_{i}}\Delta s$$

for 1≤*i*≤*N*_*p*_×*N*_*u*_, where *u*_*i*_ indicates the *i*th parameter in **u**. This allows the Jacobian to be calculated from the Lagrange multiplier. The solution strategy is to march the discretized problem for *Z* forward in arc length, and then march the discretized problem for *H* backwards in arc length. The discretized problem for *H* is (4.22) subject to (4.21).

With this computationally cheap method of calculating the Jacobian in place, we can use a simple gradient descent algorithm to update the vector of control parameters, **u**,

4.25 $${\text{u}}_{q+1}={\text{u}}_{q}-\gamma_{q}\nabla J({\text{u}}_{q}),q>0,$$

where **u**_*q*_ indicates the value of **u** after the *q*th iteration,

4.26 $$\nabla J({\text{u}}_{q})=\left[\frac{\partial J({\text{u}}_{q})}{\partial {\text{u}}_{1}},\frac{\partial J({\text{u}}_{q})}{\partial {\text{u}}_{2}},\frac{\partial J({\text{u}}_{q})}{\partial {\text{u}}_{3}}\cdots \frac{\partial J({\text{u}}_{q})}{\partial {\text{u}}_{N_{u}\times N_{p}}}\right],$$

and **u**_0_ is the initial guess of the control parameters. If *γ* is small enough, then *J*(**u**_*q*+1_)≤*J*(**u**_*q*_) ∀*q* and the sequence {*J*(**u**_0_),*J*(**u**_1_),*J*(**u**_2_),…,*J*(**u**_*q*_)} converges to a local minimum of *J*. Note that *γ* is allowed to change at each iteration. The initial guess **u**_0_ is defined using straight passes, with the feed speed at each point based on a linear, pixel-by-pixel approximation, [25].

## 5. Experimental results

In this section, the experimental results are presented. For both processes, two different types of target surface are used. The first type of target surface is simple and smooth, which allows us to evaluate the solution by calculating the average of a set of measured profiles and minimize the effect of the process noise. Moreover, the influence of the dynamics of the machine can be compensated more easily if the target surface does not have a complex shape. The second type of target surface is freeform, which provides a more challenging test of the techniques that we have developed.

### 5.1. Experimental results for waterjet milling

We began by calculating non-straight trajectories for WJM. However, during the experimental tests it was found that the dynamics of the machine had a significant influence on the actual movement of the jet for these non-straight trajectories and the compensation techniques described in [25,28] for straight trajectories were ineffective. For this reason, just the values of the feed speed were optimized for these experiments and the jet performed straight passes in the *x*-direction.

#### 5.1.1. Etching a simple surface by waterjet milling

**Figure 8.**
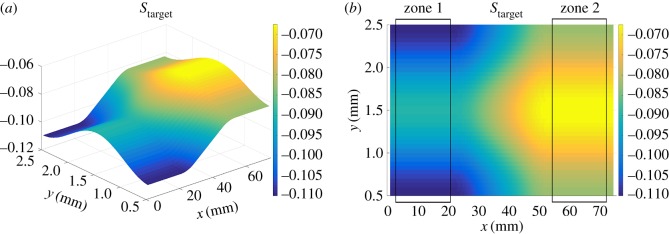
Target surface. (*a*) Three-dimensional view and (*b*) top view.

The first experiment consists of etching the target surface defined in figure 8. For this surface, the dynamics of the machine for one-dimensional trajectories can be accounted for using the technique described in [25,28]. The jet is moved using the proposed raster path method in the *x*-direction with the distance between two consecutive passes 0.2 mm.

**Figure 9.**
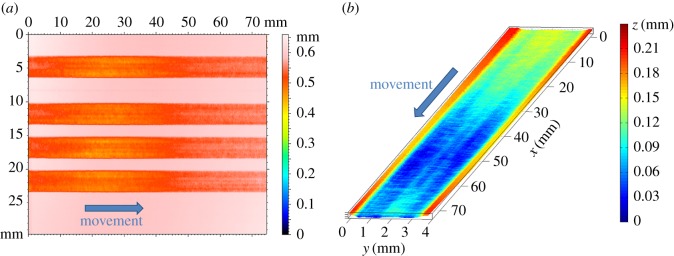
Experimental measurement of the surface etched by WJM. (*a*) Top view of four tests and (*b*) three-dimensional view of one of the tests.

**Figure 10.**
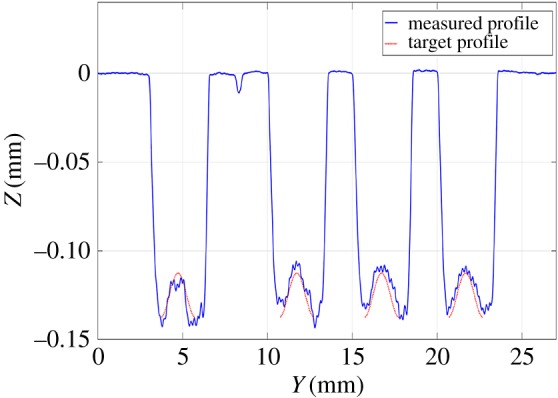
Average profiles in the *x*-direction for zone 1.

**Figure 11.**
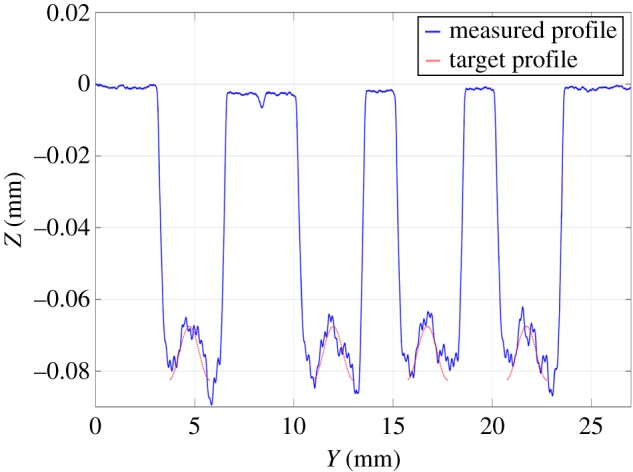
Average profiles in the *x*-direction for zone 2.

**Figure 12.**
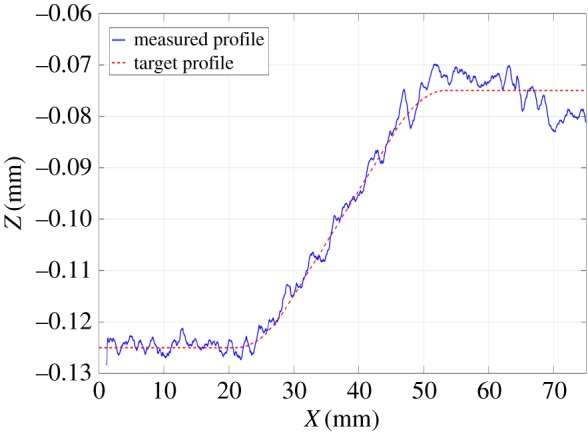
Average profiles in the *y*-direction in the centre of the surface.

The test was performed four times (figure 9*a*). Figure 9*b* shows the measured three-dimensional surface of one of these tests. The average profile in the *y*-direction was calculated for zones 1 and 2 (figure 8*b*), where the depth depends only on the position in the *y*-direction. Figures 10 and 11 show that good tracking was achieved for both sections. Finally, the profile in the *x*-direction was calculated at the centre of the surface. Figure 12 shows the average of the four profiles, and that the desired depth and shape for the different profiles was obtained. The average tracking error was calculated for each profile to evaluate the results. In this case, the value of the tracking error was between 2 and 5% for all measured profiles. Note also that the correct value of the slope was obtained with high accuracy. This is the aspect that is usually the most difficult to control when machining non-flat surfaces since the dynamics of the machine affect the actual trajectory of the jet when the feed speed is not constant.

#### 5.1.2. Etching of freeform surface by waterjet milling

**Figure 13.**
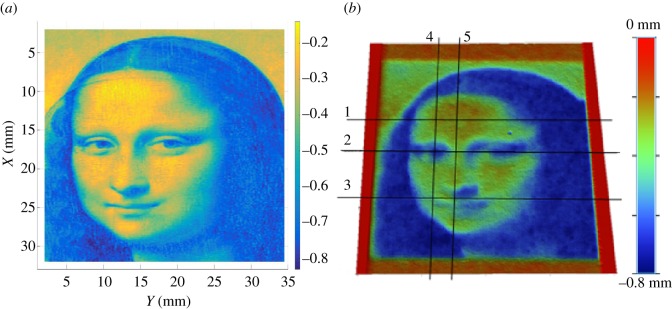
Target (*a*) and etched (*b*) by WJM freeform surfaces (size: 30× 30 mm^2^).

A more complex surface (the *Mona Lisa*) was also etched to indicate the accuracy of the proposed approach. The first step consists of generating a three-dimensional surface from an image of the painting. This was done by simply defining a different depth for each colour in the image (figure 13*a*). Adjoint optimization was used to calculate the feed speed at each point on the surface. The size of the target surface was chosen to be 30×30 mm^2^ and the same distance used between two consecutive passes, 0.2 mm. However, a different set of machining parameters was used to generate the surface in this case.

**Figure 14.**
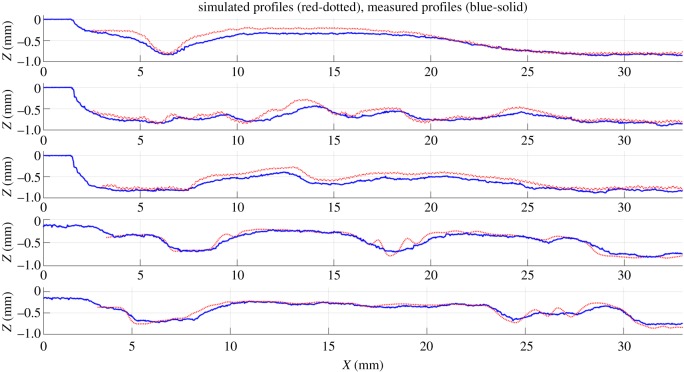
Comparison between target (dotted) and measured (solid) profiles for five different profiles defined in figure 13*b*.

The use of plain water (m_*a*_=0) and a higher value of the pressure were found to be more successful. The main reason is to avoid the influence of the dynamics of the machine. Although the dynamics can easily be compensated for ramps or simple trajectories [25,28], this compensation is very difficult when more complex trajectories are needed and the parameters of the controller are unknown. One possible strategy is to reduce the range of the feed speed so the values of the acceleration and jerk in the movement can be performed by the actual controller. When plain water is used and the pressure set to 3500 bar, the range of feed speeds needed to obtain similar values of the depth is between 100 and $400\;\mathrm{mm}\;\min^{-1}$, and the performance of the system is much less susceptible to issues with the machine controller. Note that the model needed to be recalibrated using the procedure described earlier in the paper. Figure 13*b* shows a three-dimensional image of the etched surface. A few measured profiles are compared to the simulated ones in figure 14, and the average tracking error is calculated for each one of them. The average tracking error is between 10 and 20%. These are good results for WJM, although some errors can be seen for high-frequency components. It seems that the controller is still not able to perform these rapid changes in the feed speed. The use of a faster controller or the choice of a larger, or smoother, target surface would reduce these errors.

### 5.2. Experimental results for pulsed laser ablation

The first experiment consists of etching a flat surface with four different pockets using the parallel straight lines technique. This kind of surface can be used to calculate the average profile in different zones and minimizes the effect of noise and measurement errors. The second experiment consists of etching a freeform surface. The effect of the dynamics of the machine was not important in this case since the system is fast enough to move the mirrors in the galvanometer and generate the desired trajectories of the beam. This allows us to demonstrate the advantage of using non-straight paths (with freedom to move in the *y*-direction).

#### 5.2.1. Etching a simple surface by pulsed laser ablation

**Figure 15.**
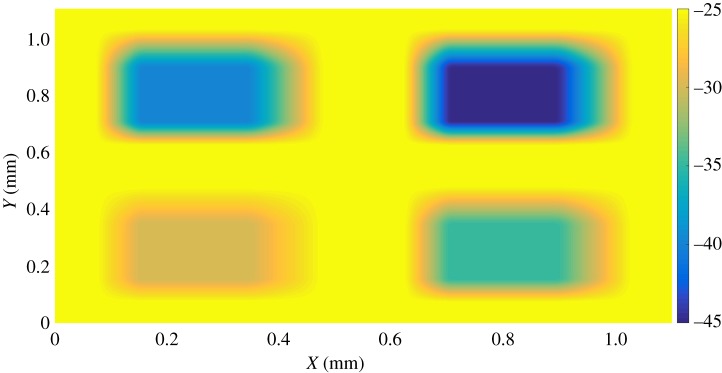
Target surface with four different pockets.

**Figure 16.**
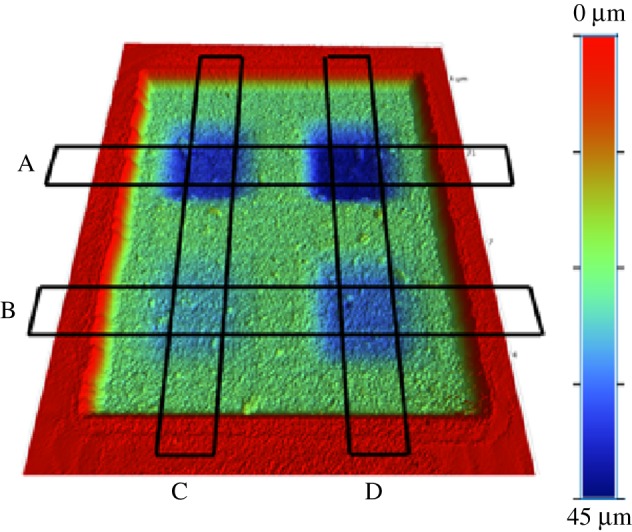
Measurement of the etched surface.

**Figure 17.**
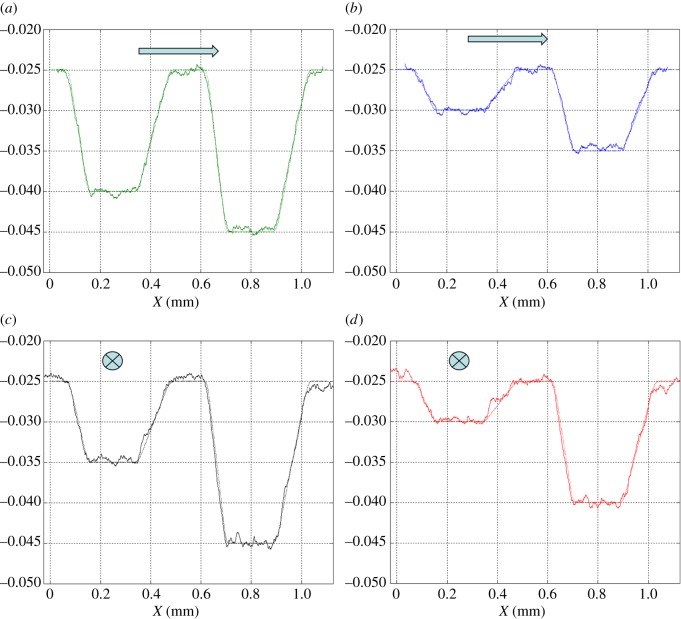
Comparison between target (dotted) and measured (solid) profiles in four different section. (*a*) Section A, (*b*) section B, (*c*) section C and (*d*) section D.

The target surface to be etched is shown in figure 15. It consists of four pockets with different depths and opposite sides of different slopes. In this particular example, it was found that the raster path technique with straight lines was good enough to mill the desired target and no improvement was seen when non-straight passes were performed. Figure 16 shows the measured surface. Four different sections were selected and the average profile calculated for each of them. A comparison between the target and measured profiles is displayed in figure 17. Not only is the desired depth achieved for the bottom of each pocket, but also the desired values of the slopes. In these four profiles, the average tracking error is around 1%.

#### 5.2.2. Etching freeform surfaces by pulsed laser ablation

**Figure 18.**
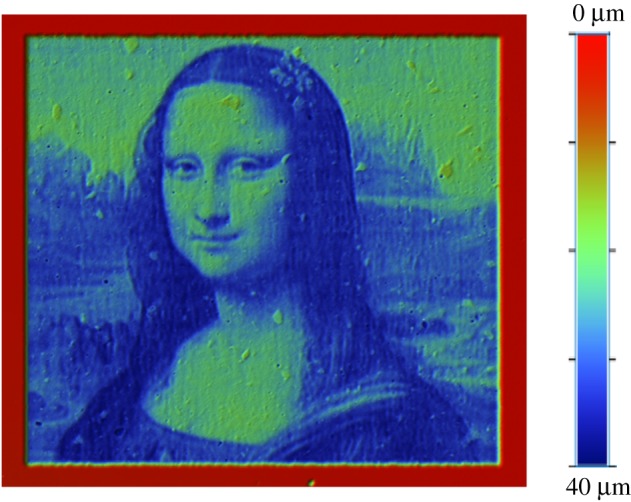
Etched *Mona Lisa* using non-straight passes.

**Figure 19.**
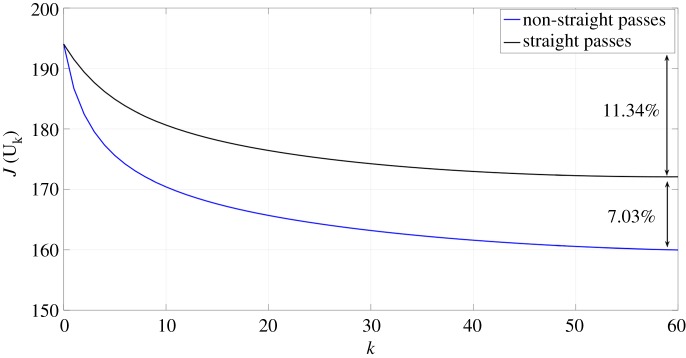
Evolution of the tracking error (*J*) of the simulated *Mona Lisa* using both strategies.

In this section, the *Mona Lisa* is again chosen as a target freeform surface. Figure 18 shows the measurement of the etched surface using non-straight passes, where the size of the target surface is 2×2.5 mm^2^. The tracking error is reduced by an additional 7% when non-straight paths are used (figure 19). The effect of the non-straight paths is not very clear on the etched surface since the shape of the target surface obtained from the painting is quite smooth and the different profiles do not have large gradients. This means that the improvement is uniformly distributed over the surface.

**Figure 20.**
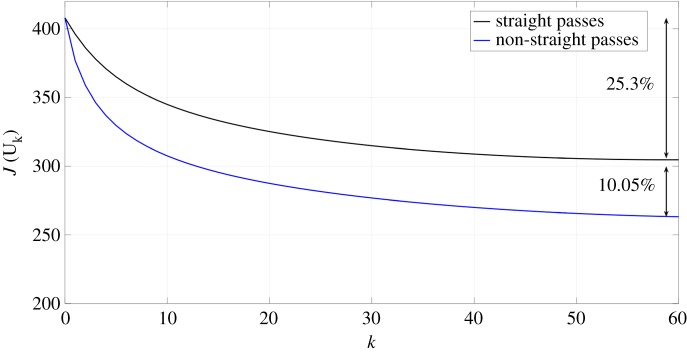
Evolution of the tracking error (*J*) for the coin when describing straight and non-straight passes are used.

**Figure 21.**
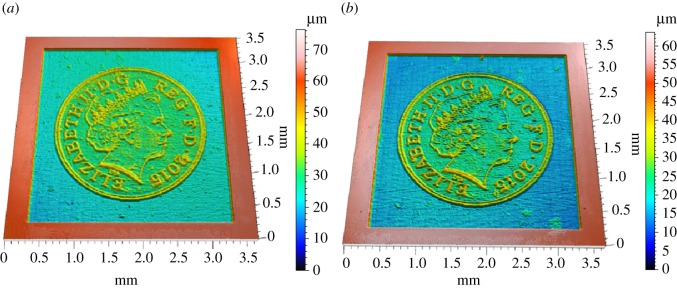
Comparison between etched surfaces using straight (*a*) and non-straight passes (*b*) (size: 3×3 mm^2^).

**Figure 22.**
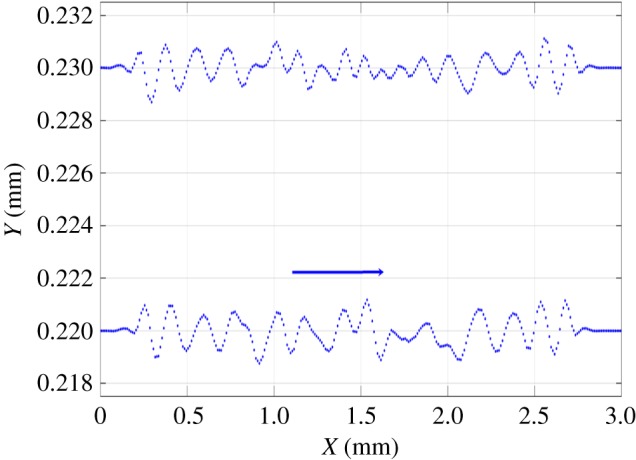
Optimized position of the points in consecutive passes.

**Figure 23.**
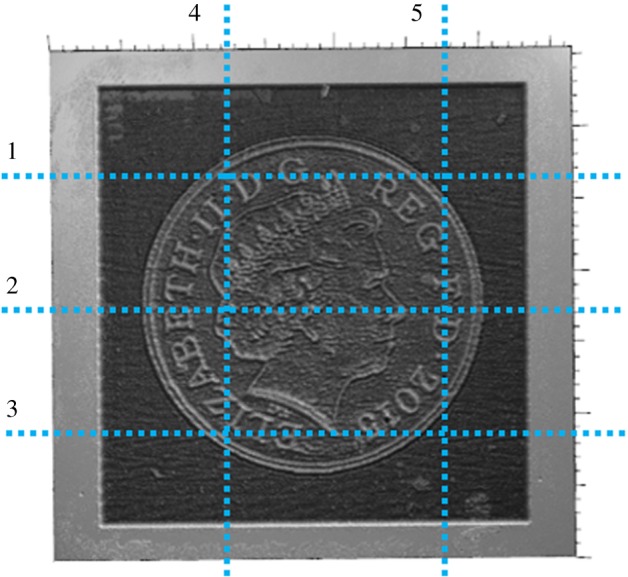
The profiles plotted in figure 24.

**Figure 24.**
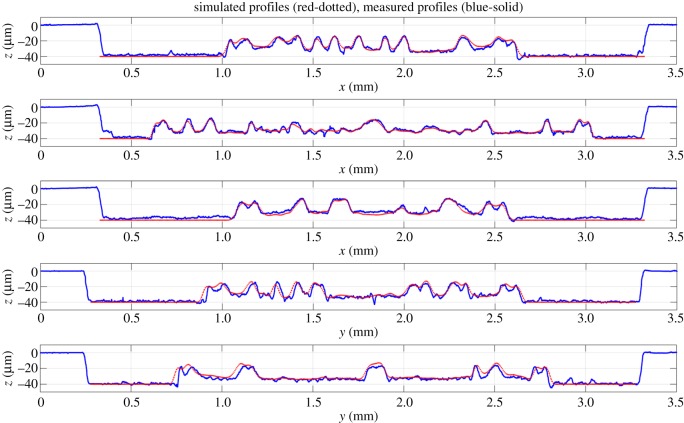
Comparison between measured and simulated profiles defined in figure 23.

**Figure 25.**
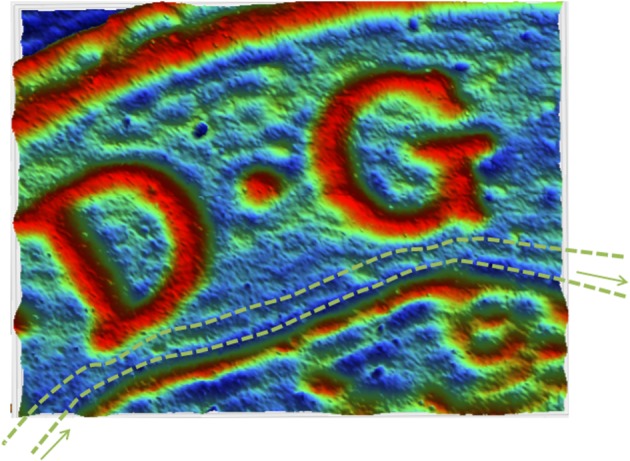
Close-up of a high-gradient zone and two consecutive non-straight passes (green dotted lines).

We also used PLA to mill a more complex target surface, with larger values of the gradient in some areas. The shape of the one penny British coin, with many steps and large values of the gradient, was found to be suitable. The adjoint optimization algorithm was applied to a 3×3 mm^2^ target surface using both straight and non-straight path strategies. The tracking error was reduced by an additional 10% when non-straight passes were used (figure 20). The initial guess of the parameters was defined by the feed speed needed to achieve the desired depth at each point on the surface without considering the influence of the other points, so the error is reduced by more than 25% compared with the pixel-by-pixel method. Figure 21 shows the measured surfaces for both approaches. The first conclusion is that non-straight passes do not improve the tracking of the etched surface on flat parts. However, edges are better resolved using this approach, and we conclude that the 10% improvement in tracking is not uniformly distributed over the surface. This effect is evident on the lines around the face or the circle of the coin. The result is improved on these parts because the beam is now free to track around edges. Figure 22 shows the trajectories of the beam in the *x*–*y* plane for two consecutive passes. These results show that the raster paths technique can be improved by allowing non-straight passes when etching freeform surfaces. Some actual profiles are compared with the target profiles in figures 23 and 24. The average tracking error in these profiles is between 4 and 8%. Finally, figure 25 shows the etched surface over a small area and the trajectory of two consecutive passes, plotted using dotted lines. This illustrates how the beam path adapts to follow the shape of sharp edges in the target surface.

## 6. Conclusion

In this paper, a new approach to solving the inverse problem for waterjet and laser etching, both modelled as dwell-time-dependent processes, has been presented. The inverse problem is defined as finding the trajectory of the beam to mill a given target surface. Our proposed algorithm for solving the inverse problem has been tested, both theoretically and experimentally, for two different energy beam processes and shown to work well. Although WJM and PLA remove workpiece material using different physical processes, a similar model can be used for both, and the same optimization algorithm applied to find the solution of the inverse problem. An adjoint optimization algorithm has been used to calculate the solution of the inverse problem. The main advantage of this algorithm is that it requires significantly less computation time than the finite difference approach to the calculation of the Jacobian matrix when a large number of parameters are estimated. In the approach presented in this paper, the complete trajectory of the beam is divided into several passes and the movement during each pass is defined by the value of the feed speed at each pixel. In addition, the usual raster paths technique is improved by allowing non-straight passes. This new feature leads to better tracking of the target surface since the trajectories can adapt to the shape of the surface. Adjoint optimization also provides a better solution than a simple pixel-by-pixel method, where the feed speed of the beam at each pixel is determined using a linear model, without taking into account the shape of the etching rate function or the overlapping of successive passes. Moreover, the use of a nonlinear model leads to better prediction of the average profiles when deep trenches or surfaces are etched.

Finally, some experimental tests have been performed for both techniques. Different target surfaces were considered and comparison with the target profiles shows that good tracking was achieved in all cases. Simple surfaces could be etched with an average tracking error between 2 and 5% using WJM, and around 1% with PLA. When freeform surfaces were etched, these values were 10–20% and 5–8%, respectively.

The limitations of the controller and the effects of noise in WJM were minimized by changing the operating parameters. However, this limitation could easily be overcome by using a faster controller.

The objective of the paper was to prove that a freeform surface can successfully be etched for both systems using the proposed algorithm. The next step will be to apply this approach to other energy beam processes, such as focused ion beam.

The approach presented here uses close to straight line passes that are all performed in the same direction. The use of passes with different orientations is being studied because this strategy may lead to a better adaptation to the shape of the target surface and reduce the tracking error. Finally, solution of this optimization problem with free paths is hard, and many subsets of the full space of possible paths (for example, spirals, Peano curves or Travelling Salesman paths) provide possible starting points for alternative surface etching strategies.

## Data accessibility

The data used for this study are available at the Dryad Digital Repository: http://dx.doi.org/10.5061/dryad.6s48n [29].
